# “I don’t think there’s necessarily a one size fits all” negotiating competing priorities in nurse shift scheduling: a qualitative study

**DOI:** 10.1186/s12912-025-03705-6

**Published:** 2025-08-11

**Authors:** Hannah Ruth Barker, Peter Griffiths, Chiara Dall’Ora

**Affiliations:** 1 University of Southampton, Southampton, UK; 2 NIHR Applied Research Collaboration Wessex, Southampton, UK

**Keywords:** Staffing and scheduling, Flexible scheduling, Shift work, Workforce, Work-life balance, Professional organizations, Nursing, Personnel staffing and scheduling

## Abstract

**Background:**

The nursing workforce globally faces significant challenges, including burnout, stress, and absenteeism, exacerbated by unsafe staffing levels and suboptimal working conditions. In England, many nursing staff express intentions to leave their roles, driven by work-life imbalance. This study explores how the preferences and constraints of nursing staff, nurse managers, and hospital directors interact to influence shift scheduling decisions within the NHS, aiming to identify strategies that reconcile individual wellbeing with organisational imperatives.

**Methods:**

This qualitative study employed framework analysis, guided by the Thomas-Kilmann Conflict Mode Instrument (TKI) to understand conflict management approaches in shift scheduling. Data were collected through semi-structured interviews with 17 nursing staff, five nurse managers, and six hospital directors across five diverse NHS Trusts in England. Interviews were conducted remotely, transcribed verbatim, and analysed to identify key themes and patterns.

**Results:**

Three primary themes were identified: Balancing Choice with Consistency, Predictability, and Flexibility; Adequate Rest and Recovery Between Shifts; and Enjoyment and Engagement at Work. The study found that collaborative and compromising conflict management approaches were most effective in preventing potential conflicts from escalating into actual conflicts. Flexible and predictable scheduling was crucial for enhancing nurse wellbeing and retention, while rigid policies often led to increased turnover and reduced morale. The study also highlighted the importance of considering external constraints, such as caring responsibilities, which can limit the effectiveness of workplace solutions.

**Conclusions:**

Effective nurse shift scheduling requires a blend of conflict management strategies, with an emphasis on collaborative and compromising approaches. By prioritising flexible scheduling and proactive communication, healthcare organisations can better support their nursing workforce, enhancing both individual wellbeing and organisational efficiency. These findings have significant implications for improving the sustainability and quality of healthcare service provision.

**Supplementary Information:**

The online version contains supplementary material available at 10.1186/s12912-025-03705-6.

## Introduction

The nursing workforce globally faces escalating challenges of burnout, stress, and absenteeism, alongside persistent concerns regarding unsafe staffing levels and suboptimal working conditions [1]. This widespread workforce depletion poses a significant threat to the sustainability and quality of healthcare service provision. In England specifically, a substantial proportion of nursing staff have expressed intentions to exit their current roles or leave the profession entirely [2]. Work-life imbalance has emerged as a predominant factor influencing departure decisions among nursing professionals [2], with established associations between unfavourable shift patterns and compromised work-life balance [3, 4].

Healthcare organisations, particularly within the National Health Service (NHS), face the complex challenge of balancing organisational imperatives for cost-efficiency through optimised rostering with the imperative to support staff wellbeing through appropriate work scheduling. This tension has become increasingly salient as evidence suggests that prioritising financial and efficiency metrics at the expense of staff considerations has contributed to declining morale and increased sickness rates among nursing personnel [5].

The relationship between shift scheduling autonomy and employee wellbeing has been established in broader occupational literature. Meta-analytical evidence indicates that flexible work arrangements incorporating greater personal choice correlate with improved physical health outcomes and reduced absenteeism [6]. Within nursing-specific contexts, empirical investigations have predominantly demonstrated positive outcomes associated with increased scheduling choice, including reductions in sickness absence, burnout rates, turnover intention, and work-life conflict [4, 7–10]. However, the literature is not entirely consistent, with some studies reporting equivocal findings regarding the relationship between scheduling autonomy and nursing staff wellbeing [11, 12].

A notable gap exists in understanding the complex interplay between competing priorities and preferences that shape shift pattern decisions across organisational hierarchies. Emmanuel et al. [3] conducted an extensive qualitative investigation revealing that nurses’ shift pattern preferences predominantly relate to non-work commitments, schedule predictability, and temporal flexibility. Similarly, recent qualitative research by Booker et al. [13] highlighted that nurses prioritise shift patterns compatible with personal circumstances, sometimes potentially compromising evidence-based fatigue management principles.

To conceptualise how preferences and constraints interact across organisational levels (nursing staff, nurse managers, and hospital directors), we employ the Thomas-Kilmann Conflict Mode Instrument (TKI) [14] as our theoretical framework. This well-established model delineates five distinct approaches to conflict management: collaborating, compromising, accommodating, avoiding, and competing. The TKI framework has been extensively utilised to analyse conflict management strategies within nursing contexts [15], providing a robust theoretical foundation for our analysis. In the current study, the application of the TKI theoretical framework gives the analysis an innovative perspective on a widely discussed topic that is rarely addressed from the lens of conflict management.

In our conceptualisation of conflict, we distinguish between two fundamental forms: potential conflict and actual conflict. Potential conflict encompasses discordant needs, priorities, or preferences that create latent conditions for disagreement without necessarily manifesting as overt discord. Actual conflict represents the explicit expression of these conflicting priorities through observable disagreement, characterised by interpersonal tension or frustration. Importantly, the presence of conflicting priorities does not inevitably precipitate actual conflict. These conceptual distinctions align with seminal conflict theory [16, 17] and have been applied to understanding workplace dynamics within nursing environments [18].

Our study aims to explore how the preferences and constraints of nursing staff, nurse managers, and hospital directors interact and collectively influence decision-making processes regarding shift patterns. Through examining these complex interactions, we seek to identify approaches that might better reconcile the potentially conflicting priorities of individual wellbeing and organisational imperatives in nursing workforce management.

## Methods

### Study design

This study adopted an interpretive qualitative approach to understand competing priorities in nurses shift scheduling across different participant groups with data analysis guided by framework analysis. Framework analysis provided a systematic and flexible approach to managing and analysing qualitative data for both inductive and deductive thematic development [19]. A subtle realist ontological position was selected acknowledging that the social world exists independently of individual perspectives while recognising that understanding is shaped by unique experiences and interpretations [20]. This allowed for interpretation of diverse perspectives that acknowledged individual and broader social experiences across participant groups. Crucially, TKI served as a guiding theoretical framework, providing concepts that informed the development of our themes and subsequent interpretation of findings, particularly in relation to understanding conflict management.

### Ethical approval

In accordance with the Declaration of Helsinki ethical approval was granted by the Health Research Authority (IRAS ID: 327884) and the University of Southampton ethics committee (ID: 81204).

### Participants

A purposive stratified sampling framework was used to ensure diversity in settings by geography and healthcare specialty. The study advertisement was distributed through the National Institute for Health and Care Research (NIHR) Research Delivery Network. Local research and Development (R&D) offices that contacted the research team were considered for inclusion. Five diverse NHS Trusts were recruited, representing the North, Midlands, East, and South of England. Two specialised in Mental Health, one in Paediatrics, and two were general NHS Foundation Trusts. Within each Trust, the R&D team or local Principal Investigator advertised the study through tailored recruitment channels. Eligible participants included nursing staff (registered nurses, nursing associates, and healthcare support workers) working shifts in inpatient settings, ward managers in inpatient settings, and hospital directors working in Human Resources or Finance. Participants self-selected to participate in the study, which means individuals with stronger opinions, available free time, or interest in the £20 voucher received as compensation for the interview time, may have been more likely to volunteer. From those who volunteered, participants were purposively approached to ensure diversity of staff roles. In our study, we ensured that paying participants was ethically sound. We respected their time and effort, promoting social good and inclusivity. Our payment scheme was designed to avoid exploitation, offering fair compensation without undue inducement. We maintained transparency about payments, fostering trust and autonomy. This approach valued participants’ contributions and supported diverse, equitable research participation.

All participants provided informed consent either online or via audio prior to data collection occurring.

### Data collection

Interviews were conducted remotely via Microsoft Teams video calls between October and December 2023. Participants joined from their preferred locations, which included workplace offices and home environments.

Semi-structured interviews were conducted with 17 nursing staff working shifts (including registered nurses and healthcare support workers), five nurse managers, and six hospital directors.

The interview guides were developed for this study based on the research objectives and are included in a supplementary file. Topics included experiences and perceptions of nurse shift patterns, typical shift patterns, satisfying and challenging aspects, ideal shift pattern factors, non-negotiables in shift patterns, and descriptions of well-being and work-life balance. No pilot testing of the interview guides was conducted, but questions were refined iteratively as the study progressed. All interviews were audio-recorded via Microsoft Teams and transcribed verbatim by HRB. Field notes were made during and after interviews to capture contextual information and non-verbal cues. Data collection continued until saturation was reached in nursing staff responses, with no new themes identified. The group interview format for hospital directors was deliberately chosen despite the potential for social desirability bias. This approach aimed to foster more candid exchanges among directors from different trusts, with the expectation that peer observations would encourage greater openness and generate richer insights.

### Research team and reflexivity

The primary researcher (HRB) conducted all interviews. HRB is a female researcher with previous experience in qualitative data collection and analysis. The researcher had no established relationships with participants prior to study commencement. Participants were informed only that the researcher had a professional interest in nurse shift patterns. The researcher introduced herself, her credentials, and the purpose of the research during recruitment. Throughout the data collection process, HRB maintained a reflective journal to enhance awareness of potential personal biases that might influence the interview process or data interpretation.

### Data analysis

Framework analysis was employed following the approach described by Ward et al. [19]. The analysis process involved:

1. Transcription and familiarisation with the data through repeated reading.
2. Initial coding of transcripts.
3. Development of a working analytical framework.
4. Application of the framework to all data.
5. Charting data into the framework matrix.
6. Interpretation of the data.

Initially, themes were organised deductively according to known attributes related to nurse shift scheduling (shift patterns, rostering, pay, wellbeing, and life outside of work). However, after team discussion, these themes and sub-themes were inductively reorganised into three overarching themes.

Three researchers (HRB, CDO, and PG) were involved in data analysis. Regular meetings were held to discuss code and theme development, enhancing analytical rigour. HRB led the data analysis, maintaining a clear audit trail that preserved the connection between codes, themes, and original transcripts. This approach facilitated collaborative theme development [21].

### Trustworthiness and transparency

To ensure trustworthiness, criteria from Lincoln and Guba [22] were followed:

#### Transferability

Purposive sampling techniques were used to select diverse NHS Trusts and participant roles. Socio-demographic data were collected (Tables 1 and 2), showing variety in years with current employer and years in current role. However, gender and country of training were relatively homogenous, but this is reflective of the national workforce. In the NHS, nurses are predominantly female (89.4%), and the majority have English nationality (71.3%) [23, 24].

#### Reflexivity

HRB maintained a reflective journal throughout data collection and analysis to acknowledge potential personal biases and other biases such as recruitment and social desirability bias. These reflections enhanced transparency in the findings.

#### Confirmability

Multiple researchers (CDO and PG) discussed code and theme development during regular meetings. The coding structure evolved through collaborative analysis, with a clear audit trail preserving the connection to original data. A coding tree of the agreed themes is available in a supplementary file.

#### Credibility

Nurse leaders across the five participating Trusts reviewed the study findings, confirming that results resonated with their experiences and perspectives. No formal participant checking of transcripts was conducted.

To support transparency, the consolidated criteria for reporting qualitative research (COREQ) checklist [25] was completed and is available as supplementary file.

## Findings

Individual interviews with nursing staff and nurse managers lasted between 20 and 55 min. Hospital directors participated in one group interview lasting 55 min. Socio-demographic data were collected. While participants showed varied lengths of time with their current employer and in their current role, as detailed in Tables 1 and 2, gender and country of training were relatively consistent. This consistency reflects the composition of the national nursing workforce; NHS nurses are overwhelmingly female (89.4%) and largely of English nationality (71.3%) [23, 24].

**Table 1:** Nurse staff and nurse managers characteristics (*n* = 22)

**Sex**		**Country of Training**	
Female	20	UK	14
Male	2	Non-UK	4
**NHS Setting**		NA	4
Community & Mental Health	10	**Work Pattern**	
General Hospital	10	Full Time	21
Paediatrics	2	Part Time	1
		**Years with Current Employer**	
**Job Role**		0 to 3	4
Senior Manager	1	4 to 6	7
Ward Manager	3	7 to 9	3
Nurse Practitioner/Specialist	2	9 +	8
Nurse Team Leader/Senior Staff Nurse	3	**Years in Current Role**	
Staff Nurse	6	1	6
Nurse associate	3	2	6
Health Care Support Worker	4	3	4
		4	1
		Over 5	5

**Table 2:** NHS director characteristics (*n* = 6)

**Years with Current Employer**	
0 to 3	2
4 to 6	0
7 to 9	0
9 +	4
**Years in Current Role**	
1	1
2	2
3	0
4	1
Over 5	2
**NHS Setting**	
Community & Mental Health	2
General Hospital	2
Paediatrics	2

This study identified three key themes related to nurse shift scheduling preferences and their impact on wellbeing and organisational functioning: (1) Balancing Choice with Consistency, Predictability and Flexibility; (2) Adequate Rest and Recovery Between Shifts; and (3) Enjoyment and Engagement at Work. These themes are analysed through the TKI to understand how different conflict resolution approaches—competing, collaborating, compromising, avoiding, and accommodating—manifest in nurse shift scheduling contexts.

### Theme 1: Balancing choice with consistency, predictability and flexibility

Nursing staff valued having a consistent shift pattern that aligned with their individual preferences while also allowing for predictability and flexibility in scheduling. Three key attributes were identified as essential components of effective shift scheduling. Within this theme, various TKI conflict management modes were identified as stakeholders navigated competing priorities:

#### Consistency in shift patterns

Nurses strongly emphasised the need for consistent shift patterns rather than “sporadic” (ID8; ID10) or “random” (ID9) shifts. Mixed day and night shifts were particularly problematic, causing disorientation and difficulty in planning personal activities: “*It’s all shuffled and there’s no routine in your life…When you work when you have a routine in your life… I go out to my friends… I go to gym…I reboot*“ (ID7). What constituted a “consistent” schedule varied based on individual circumstances. For some, consistency meant specific days off for childcare, while for others, it meant predominantly working night shifts: *“I actually was doing most night shifts when I returned to work after maternity leave because my husband was doing day shifts*,* was working nine to five. So I had to do night shifts just to balance who takes care of our baby”* (ID30). Failure to accommodate these individual consistency needs led to staff turnover, as one nurse manager noted: “*They lost about 3 qualified nurses to a different trust because they weren’t given…set days for…childcare*” (NM14).

When nurses strongly advocated for consistent shift patterns that met their personal needs, they demonstrated a competing approach, prioritising their own concerns over organisational flexibility. Organisations that failed to accommodate these needs experienced increased turnover, suggesting that an avoiding approach to addressing nurses’ scheduling concerns was counterproductive. Successful nurse retention required managers to adopt at least a compromising approach to scheduling conflicts.

#### Predictability in scheduling

All stakeholders—nurses, managers, and directors—valued rosters being released well in advance. Directors recognised operational benefits: “*The earlier that you can actually get them approved*,* signed off*,* any vacant shifts uploaded to NHSP and filled with bank rather than having to go out to agency*,* is a lot better*” (D4). For nursing staff, advance notice facilitated better work-life planning: “*If you know what days you’re working and which days you’re off*,* then it’s easier for you to plan*” (ID2). Fairness was identified as a shared value across all levels, with unpredictable, last-minute scheduling perceived as inherently unfair: “*We have a lot of unfairness around rostering at those periods of time (i.e.*,* Christmas*,* holidays) because they (i.e. the rosters) are done at such short notice*” (D3). Directors characterised fairness as a “win-win” that benefited both staff and the organisation, while nurses acknowledged that fairness sometimes required accepting less desirable shifts: “*If I could choose not to work weekends*,* I probably would. But then that means someone else would have to work them*,* and that’s not fair*” (ID8).

Predictability in scheduling represented a collaborating approach where all stakeholders’ needs aligned. Both organisational efficiency goals and nurses’ personal planning needs were served by advance roster publication, creating a true “win-win.” When nurses acknowledged the necessity of working some undesirable shifts for fairness, they demonstrated a compromising mode, showing willingness to partially sacrifice their preferences to ensure equitable distribution of shifts among colleagues.

#### Flexibility in management approach

Nurse managers prioritised accommodating staff preferences to maintain workforce satisfaction: “*If I make sure everyone’s kind of like happy with the off duty that anxieties aren’t attached to that. Everyone’s fairly happy then you’ve half the battle*” (NM14). This required individualised approaches to staff management: “*It’s about assessing the individual. I don’t think there is a blanket rule*” (NM3). Managers observed that accommodating staff preferences improved attendance and retention: “*Once you get to know your workforce…you can play what they want and then they’re going to attend…my sickness went from double figures percentage to like 2.3%*” (NM14). Flexible working arrangements enhanced staff satisfaction and work-life balance: “*I do have agreed flexible working. Suits me and it suits my needs. I’m able to go to the gym on a Monday night… I’m able to see family more…I’m able to*,* you know*,* go on holiday*” (ID21). However, managers faced constraints in providing flexibility due to competing demands: “*When we do the off duty*,* we have to make sure the ward is covered first and…only then*,* we can honour those hours like you know the additional hours for the learning opportunity*” (NM11). Directors acknowledged system-wide challenges: “*Now that the NHS are really pushing flexible working it’s really quite difficult to do that… Especially one of our significant challenges is term-time only contracts… Because if you have maybe nine nurses on one ward it’s difficult to grant them all term time only contracts*,* so it’s about finding that balance*” (D2). Staff experienced frustration when managers were inflexible to reasonable requests: “*Doing 2 long days were too difficult for me in a row because I was hardly sleeping… I asked them if I’m working Monday a long day*,* can I have a day off on Tuesday and work again on Wednesday… But it wasn’t*,* it was not possible*” (ID7). Directors’ priorities focused primarily on safety and budget concerns, with patient safety as “*the top priority*” (M4), followed by efforts to save “*money and make services more efficient*” (M4). The nursing shortage had forced organisations to become more accommodating of staff preferences: “*We didn’t have night shifts only contracts*,* it was not allowed*,* but now we have softened because we’ve realised*,* you know*,* we’ve gotta go with what individuals want rather than it to be what the organisation wants. We’re in a buyers’ market and that is what’s pushing our behaviours*” (D3).

Successful nurse managers employed an accommodating approach when creating rosters, prioritising staff preferences to improve satisfaction and reduce turnover. This approach recognised that addressing nurses’ needs yielded organisational benefits through reduced sickness absence and improved retention. When managers’ responses to staff scheduling requests were inflexible, they demonstrated an avoiding conflict management style that generated frustration among staff. The healthcare labour shortage has shifted organisational approaches from competing (enforcing organisational policies) to more accommodating strategies (accepting individual scheduling preferences), reflecting the “buyers’ market” reality where nurses have increased bargaining power.

### Theme 2: Adequate rest and recovery between shifts

All stakeholders recognised that nursing work is emotionally, psychologically, and physically demanding, making sufficient rest between shifts essential. Actual conflict arose when rosters prevented adequate recovery, particularly when: Shift finish times were too late to allow proper unwinding before the next shift; insufficient rest was provided after intensive work periods; too many shifts were scheduled consecutively: “*That would mean that you have something crazy like 6 shifts together without a break*” (ID9). The availability of days off was highly valued by staff working long shifts: “*The days off*,* I think by doing long days it means I get more days off so I find that better for a work life balance*” (ID17). Recovery between shifts was crucial for adequate sleep; emotional recuperation; managing personal responsibilities; social connection with friends and family. Effective shift patterns positively impacted wellbeing: “*My wellbeing*,* I would say*,* is good when my shifts are within a sensible sort of pattern during the week… when I have weeks…when I had both weekends either side of three shifts*,* then a training day*,* then that’s when my wellbeing sort of takes a bit of a toll and I feel like I don’t have enough time to recover properly*” (ID9). Even with flexible working arrangements, some nurses—particularly those with childcare responsibilities—still struggled: “*So I do 3 long shifts a week… it tends to be 2 long days in the week and then… always a shift at the weekend*,* whether that’s a day or a night…. It’s not working for me at all at the minute. Just work life balance is horrible basically*” (ID6). Earlier finishing times for long day shifts were identified as a significant factor in improving work-life balance: “*Your adrenaline is going and then you get to like 7:00pm… and you’re just like mentally drained. But then you still have two hours more to go*,* so it feels like it just goes on and on some days*” (ID13). Earlier finish times provided valuable recovery time: “*The times where you know if it actually finishes at half seven. And you go home… You’ll get a couple of hours in the evening before you have to get up the next day*” (ID21). Some managers recognised that earlier finish times could align with organisational priorities: “*The end time of a shift pattern is a big difference in how people feel… my argument to get that time and pushed back is actually we save on budget*” (NM1).

Rest and recovery issues revealed tensions between organisational staffing needs and nurses’ wellbeing requirements. When managers scheduled consecutive shifts without adequate breaks, they employed a competing approach that prioritised organisational coverage needs over individual recovery requirements. Nurses who accepted long shifts in exchange for more days off demonstrated a Compromising strategy, trading intensity for extended recovery periods. The nurse manager who advocated for earlier finish times by connecting it to budget savings exemplified a collaborating approach, seeking solutions that simultaneously addressed both staff wellbeing and organisational priorities. For nurses with ongoing work-life balance challenges despite flexible scheduling, the absence of viable solutions suggested an Avoiding conflict resolution style from both organisational and individual perspectives.

### Theme 3: Enjoyment and engagement at work

Nursing staff valued meaningful patient interactions and team collaboration: “*Interacting with the patient more…spending more time with them and learning more about them…And actually seeing…people… my manager…like doctors…just knowing how the ward works…it’s much*,* much better*” (ID18). These priorities were more consistently met among nurses working shorter daytime shifts compared to those on long day or night shifts. Nurse managers emphasised the importance of team cohesion: “*Everybody knows…we’re gonna be here for this long together. And we only have three people on shift which I think kind of reinforces that you need to be a lot more of a tighter team because then if one person’s upset or one person disrupts something that can cause quite a big ripple effect*” (NM32). Nurses also acknowledged the trade-offs in shift work to achieve job satisfaction: “*There’s trade-offs*,* isn’t there all the time between*,* OK*,* so I’ll work longer*,* but then I have more time off. So that there’s never. I don’t think there’s necessarily kind of a one size fits all*,* but it’s about kind of balancing the trade offs to a point where actually the job is satisfying”* (ID10). Conflicts were identified around shift start/end times and handover periods. While a 30-minute handover was considered necessary for thorough communication, this extended either the start or end time of shifts. Even with scheduled 15-minute handovers, these often ran longer, resulting in delayed shift endings. A common shift pattern described was 7am to 7:30pm (days) or 7pm to 7:30am (nights), with 30-minute handovers. Some managers provided additional support through allocating “time off the floor” for administrative work: “*It makes them feel appreciated about you know what they’re doing because it’s not normal for a band 5 staff nurse to have a management day*,* and I think*,* well*,* they need it though*” (NM14). They also offered career development opportunities through personalised conversations: “*Have you thought about this course or that training opportunity*” (NM32). However, organisational wellbeing initiatives were often perceived as inaccessible to shift workers: “*Health and wellbeing department and they put on like lovely events…and they forget about shift workers… I can’t leave the unit for an hour and go on a lovely midday walk with you. You know*,* we’ve got a staff gym just opened. It’s open four till seven. I don’t finish my shift until half seven*” (ID6).

The tension between adequate handover time and shift length illustrated a compromising approach where neither optimal communication nor ideal shift length could be fully achieved. Nurses working long shifts or nights experienced reduced opportunities for meaningful patient and team interactions, representing an avoiding approach to addressing this conflict between shift structure and engagement quality. Managers who allocated “time off the floor” for administrative work demonstrated an accommodating style that recognised nurses’ professional development needs despite organisational pressures. The disconnect between organisational wellbeing initiatives and shift workers’ realities reflected an avoiding conflict management approach from the organisational perspective, where the fundamental incompatibility between standard business hours and shift work scheduling remained unaddressed.

In summary, the analysis of nurse shift scheduling through the TKI framework reveals how different conflict management approaches shaped the experiences of nursing staff, nurse managers, and hospital directors:

Competing approaches were observed when:

- Nurses strongly advocated for personal scheduling preferences.
- Organisations strictly enforced policies without accommodation.
- Managers prioritised coverage over adequate rest periods.

Collaborating approaches were identified in:

- Advance roster publication benefiting both staff planning and organisational efficiency.
- Managers linking earlier shift finish times to budget savings.

Compromising strategies appeared when:

- Nurses accepted working undesirable shifts for fairness.
- Staff traded long shift intensity for more days off.
- Balancing handover time against shift length.

Avoiding conflict styles were evident in:

- Organisations failing to address nurses’ scheduling concerns.
- Persistent work-life balance challenges without viable solutions.
- Wellbeing initiatives that excluded shift workers.

Accommodating approaches were demonstrated by:

- Managers prioritising staff preferences to improve satisfaction and retention.
- Organisations adapting policies in response to nursing shortages.
- Providing “time off the floor” for professional development.

This analysis reveals that effective nurse shift scheduling requires a sophisticated blend of conflict management approaches, with greater emphasis on collaborating and compromising strategies to balance individual needs with organisational requirements. The transition from rigid organisational policies to more accommodating approaches reflects the evolving power dynamics in healthcare employment, with nurses gaining leverage in a competitive labour market.

## Discussion

Our study was the first to investigate how different priorities of nursing staff, nurse managers, and hospital directors interact and shape nurse shift patterns. Using the Thomas-Kilmann Conflict Mode Instrument [14], we identified patterns of potential conflicts, actual conflicts, and occasional compatibilities across three primary domains of shift scheduling concerns. Despite interviewing a variety of nursing staff and nurse managers, we developed three overarching themes influencing nursing staffs’ decision making in shift scheduling: Balancing Choice with Consistency, Flexibility, and Predictability; Adequate Rest and Recovery between Shifts; and Enjoyment and Engagement at Work.

Applying the TKI framework [14], our findings demonstrated how conflict management approaches influenced scheduling outcomes. Collaborative and compromising approaches, characterised by high concern for both self and others, prevented potential conflicts from escalating. For instance, nurses’ preferences could be met through collaborative compromises that simultaneously supported the organisation’s staff retention goals. This aligns with Pondy’s [17] model of conflict, suggesting latent conflicts become manifested conflicts when effective management strategies are absent.

In contrast, avoiding (low concern for self and others), or competing (high concern for self, low concern for others) approaches led to actual conflict and nurse attrition. Accommodating approaches (low concern for self, high for others) were “too simplistic” for complex multi-stakeholder priorities in scheduling, often leaving one party’s needs unmet. When nurses continuously accommodated organisational needs without reciprocity, their work-life balance and wellbeing suffered, consistent with evidence on work time control [26] and links between autonomy and wellbeing outcomes.

Recent qualitative work highlights features valued by nurses in shift scheduling such as roster consistency, work time control, and flexibility [3], and preference acknowledgement [27]. Our research uniquely interprets how these overlapping principles interact using the TKI.

In the theme “Balancing Choice”, consistency, flexibility, and predictability fostered collaborative conflict management [14]. Consistency set clear expectations as a foundation for mutually beneficial solutions. Flexibility from nursing staff and managers allowed adaptation and responsiveness to less desirable shift patterns and staff individual needs, exemplifying compromise where both parties achieve partial satisfaction. Effective communication and awareness between staff groups – core principles of constructive conflict management – could manage expectations. Nursing staff could expect some consistency in their shift patterns for their individual needs (addressing their concerns), but also accept give-and-take within organisational constraints (addressing others’ concerns). Predictability helped nurse managers make less desirable shifts more acceptable through reducing uncertainty and providing fair processes. This theme revealed an important paradox in nursing shift scheduling preferences: they simultaneously desire individual autonomy (choice and flexibility) and structural reliability (consistency and predictability). Our findings suggest that effective scheduling must address both of these needs.

Our research also expands theoretical understanding of choice in nurse shift scheduling and wellbeing. Our study consistently showed choice benefitted perceptions of wellbeing and work-life balance, suggesting people feel better with more perceived choice. These results align with most research exploring nursing staff choice in shift scheduling and wellbeing outcomes (i.e., more job satisfaction, less sickness absence, more work-life balance, less fatigue, less burnout) demonstrating positive associations between choice for shift scheduling and wellbeing outcomes [4, 7, 10, 28, 29]. However, our study revealed an important nuance: external constraints, like caring responsibilities, sometimes meant nurses remained “stuck” regarding work-life balance despite offered choice. These findings add novel insight to work-life balance theory by highlighting workplace solutions’ limitations when external factors constrain true choice. A recent scoping review noted similar findings suggesting job satisfaction may be sacrificed to balance personal demands [27]. This illustrates the TKI accommodating mode, where individuals sacrifice their concerns to satisfy others’—in this case, external responsibilities. This theoretical insight may explain why some studies on choice for nurses in shift scheduling and perceived wellbeing that did not adjust for lifestyle constraints outside of work showed little effect [11].

Our results showed the importance of rest and recovery between shifts and enjoyment and engagement at work. For example, actual conflict occurred—in terms of Pondy’s [17] manifest conflict stage—when there was not enough rest for nursing staff between shifts and too many shifts were bundled together. Many nurses felt long days facilitated better rest and downtime. Our data indicates nurses recognise that wellbeing impacts on their ability to provide quality care sustainably, fostering job satisfaction—integrating professional values with personal wellbeing. While one qualitative study expressed concern that complete autonomy in shift choice could prioritise other needs over individual well-being [13], our “Rest and Recovery between Shifts” theme showed how crucial rest and recovery were for nurses’ work and personal lives.

However, similar to other research [30, 31], our findings also indicated a shortfall in positive team dynamics, handover, and training opportunities with long day shift patterns. Our study uniquely showed that although valuing these elements, nurses frequently prioritised maintaining long day shift patterns for work-life balance. This represents a theoretical compromise where one professional value (team cohesion) is partially sacrificed for another (work-life balance), consistent with the compromising mode in the TKI framework. These attitudes may reflect a broader societal shift towards prioritising well-being and work-life balance [6]. Hospitals need to be ready to respond to these changing priorities. For example, increased nurse advocacy for shift scheduling needs could shift power dynamics, prompting more collaborative scheduling processes. When nurse managers provided “time off the floor” for training, it supported quality care delivery. This represents an integration of competing priorities rather than compromising—fully meeting the individual’s development needs and organisation’s service needs, exemplifying the collaborative mode of conflict management.

Within the three overarching themes, we observed examples of when potential for conflict remained as such (latent conflict in Pondy’s [17] terms), with successful compromises from different staff groups, and when it became actual conflict (manifest conflict). The most obvious trend was when there was no flexibility at all—when complete rigidity on the priorities of different staff groups (competing approach) resulted in actual conflict. For example, if only rostering targets were considered in shift scheduling decisions, or if an individual with a non-negotiable need faced inflexibility, actual conflict emerged. Sometimes a small tweak like an earlier shift finish or specific shift pattern (e.g. no consecutive long day shifts), was enough – representing compromise. However, gaining agreement for small changes in the organisation could prove difficult, suggesting organisational resistance to change [32].

Even when compatibilities existed between nursing staff and the organisation, these were underpinned by different priorities, creating latent conflict. For example, directors embraced flexibility while acknowledging challenges but tended to be driven by operational factors, such as reducing turnover, rather than being driven by staff wellbeing effects. These different priorities and constraints perpetuated potential conflicts across the organisation, necessitating compromises to avoid actual conflict, maintain service delivery and prevent unmanageable nursing staff turnover. This aligns with Pondy’s [17] cyclical view of organisational conflict, where underlying structural factors and differing priorities ensure latent conflicts persist even after manifest conflicts are resolved.

Our study showed that each staff group’s understanding and upholding of the other staff groups’ priorities to an acceptable level helped maintain conflicts as potential, rather than overt. For example, nurse managers noted improved staff retention and reduced sickness absence when balancing nurses’ preferred shift patterns with ward staffing requirements; nurses cited leaving jobs when flexibility and roster balancing were absent. Effective communication that raised awareness and adjusted expectations and fairness facilitated this flexibility—core components of both collaborative and compromising conflict management approaches.

Our research reveals significant implications for improving shift scheduling across all levels of nursing leadership and hospital management. Effective communication is essential for all stakeholders and nursing staff benefit from clearly advocating their scheduling needs while remaining open to negotiation (the essence of the compromising mode). Where locally appropriate, nursing staff can request flexible scheduling options aligning with personal circumstances and wellbeing.

Nurse managers should prioritise implementing flexible working arrangements and systematic approaches for gathering and responding to shift pattern feedback. The theoretical basis for this recommendation is that collaborative approaches—which seek to fully satisfy both parties’ concerns—produce more sustainable solutions than competing or avoiding approaches when addressing complex scheduling conflicts. Our findings suggest particular attention to intensive schedules, such as four consecutive long days, with consideration of modifications like replacing the fourth long day with a shorter shift—a compromise that potentially improves both wellbeing and retention.

Hospital directors should reframe staff scheduling preferences as an organisational value not merely a retention compromise. This perspective shift recognises flexible scheduling as critical best practice. Specific scheduling modifications—such as earlier finishing times for long shifts and adequate rest periods—can significantly enhance nurse wellbeing, reducing fatigue and burnout.

At the systems level, our findings suggest that organisational policies proactively preventing potential conflicts—such as extending current NHS guidance beyond the six-week advance publishing standard—create conditions where collaborative approaches can flourish. Earlier roster publication demonstrably benefits staff work-life balance while affording managers more time to address roster gaps proactively, a collaborative solution meeting both individual and organisational needs.

Through the theoretical lens of conflict management theory, our research provides a framework for understanding not just preferred scheduling practices, but why certain approaches succeed while others fail, and how different conflict management strategies can transform potential conflicts into constructive outcomes for both individuals and organisations.

### Limitations

As Burr [33] suggests, participants’ awareness of being recorded and interacting with an unfamiliar researcher may have influenced their willingness to disclose complete and unfiltered perspectives. This social desirability bias potentially moderated responses, particularly when discussing sensitive topics such as disagreements with organisational policies or interpersonal conflicts with colleagues. To mitigate this limitation, the primary researcher leveraged extensive interviewing experience to establish rapport and create psychologically safe environments conducive to authentic disclosure. Furthermore, methodological rigour was enhanced through systematic reflective journaling, transparent analytical processes, and adherence to established trustworthiness criteria [22] throughout data collection and analysis phases.

A further limitation of this study is its reliance on perceptions rather than objective behavioural observations. The data refer to perceptions and are not triangulated with direct observation or documentary analysis. While our findings provide valuable insights into perceived priorities and constraints regarding shift scheduling across organisational hierarchies, they do not capture actual decision-making behaviours and choices enacted by nursing staff, nurse managers, and hospital directors. This distinction between perception and behaviour is particularly relevant in organisational contexts where institutional constraints may create discrepancies between expressed values and implemented practices.

The potential impact of regional variations in NHS Trust policies represents another limitation affecting the transferability of our findings. While our purposive sampling strategy incorporated geographical diversity across five NHS Trusts in England, organisational cultures, leadership approaches, and policy implementations inevitably differ across healthcare settings. Moreover, the findings may have limited transferability to international healthcare contexts with fundamentally different workforce structures, regulatory frameworks, or cultural orientations toward work-life balance. As is characteristic of qualitative inquiry, this research does not claim universal generalisability but rather offers contextually situated insights that may inform similar settings with appropriate adaptational considerations.

## Conclusions

Globally health care systems are losing nursing staff due to poor work-life balance. Our research points towards the importance of choice for nursing staff in shift scheduling to promote wellbeing and work-life balance. Our study shows that shift scheduling is a contentious issue and is a point of potential and actual conflict between the organisation and nursing staff due to different priorities and constraints. The study reveals that collaborative and compromising approaches are most effective in preventing potential conflicts from escalating into actual conflicts. When nurses’ preferences are reasonably met through collaborative compromises, both individual wellbeing and organisational efficiency are supported. Conversely, rigid scheduling policies and competing approaches often lead to actual conflicts, resulting in increased turnover and reduced staff morale. Our research also expands the theoretical understanding of choice in shift scheduling and its impact on nurse wellbeing. While choice generally correlates with positive wellbeing outcomes, external constraints such as caring responsibilities can limit the effectiveness of workplace solutions. This nuance highlights the need for a more holistic approach to scheduling that considers both professional and personal factors. In conclusion, effective nurse shift scheduling requires a sophisticated blend of conflict management strategies, with greater emphasis on collaborative and compromising approaches. By prioritising flexible scheduling and proactive communication, healthcare organisations can better support their nursing workforce, ultimately enhancing the quality and sustainability of healthcare service provision.

## Supplementary Information

Below is the link to the electronic supplementary material.

Supplementary Material 1

Supplementary Material 2

## Data Availability

A coding tree of the themes is provided within the supplementary information files.The qualitative data generated during this study are not publicly available due to ethical concerns related to participant privacy and confidentiality. The Participant Information Sheet did not include a statement regarding the public sharing of raw data. Anonymised excerpts are presented in the manuscript to illustrate the findings.
