# Supplementary material for: “I don’t think there’s necessarily a one size fits all” negotiating competing priorities in nurse shift scheduling: a qualitative study
Source: BMC Nurs. 2025 Aug 11;24:1048. doi: 10.1186/s12912-025-03705-6 (PMC12337461; doi:10.1186/s12912-025-03705-6)
Supplement: Supplementary file 1 — Supplementary Material 1 [file 12912_2025_3705_MOESM1_ESM.docx]

Negotiating competing priorities in nurse shift scheduling: a qualitative study

| **Meta Theme** | **Theme** | **Codes** |
| --- | --- | --- |
| 1. Conflicting priorities for nurse shift scheduling between staff groups (Nursing Staff, Nurse Managers and Hospital Directors) created potential or actual conflict.  2  3. | Balancing Choice with Consistency, Predictability and Flexibility | Nursing staff valued consistency in shift patterns to plan their lives outside work, but there were conflicting priorities between staff and managers. Predictability in schedules released in advance was important for planning and fairness. Flexibility from managers to accommodate staff preferences was valued but constrained by staffing and service needs. |
|  | Adequate Rest and Recovery Between Shifts | Sufficient rest between shifts was crucial due to the physically and emotionally demanding nature of nursing work. Recovery time influenced work-life balance and wellbeing. |
|  | e. Enjoyment and Engagement at Work | Nurses prioritised spending meaningful time with patients and the interdisciplinary team. Conflicts existed around shift start/end times and handover length, impacting wellbeing |
