# Supplementary material for: “I don’t think there’s necessarily a one size fits all” negotiating competing priorities in nurse shift scheduling: a qualitative study
Source: BMC Nurs. 2025 Aug 11;24:1048. doi: 10.1186/s12912-025-03705-6 (PMC12337461; doi:10.1186/s12912-025-03705-6)
Supplement: Supplementary file 2 — Supplementary Material 2 [file 12912_2025_3705_MOESM2_ESM.docx]

**Supplementary Material**

**1: Example Interview Topic Guide**

*NB: This is an example of the interview topic guide used during the Improving Shifts for Nursing Staff Qualitative Study. This topic guide was used for Nursing Staff in November 2023 to January 2024.*

Overall Study Aim: Our study aims to explore how the preferences and constraints of nursing staff, nurse managers, and hospital directors interact and collectively influence decision-making processes regarding shift patterns.

Opening

- Welcome participants and introduce yourself as the researcher (including name, job role and organisation).
- Ask whether the participant has any questions before we begin.
- Remind participants that they are free to withdraw at any time, and for up to 24 hours following this interview.
- Remind participants that no personal information (such as their name or employer) will be shared with anyone outside the research team.
- Remind the participant that the interview is being recorded and gain consent.
- State that the estimated interview time will be 60 minutes.
- Outline the purpose of the study and aims of today’s interview.

Main discussion

- What does your typical shift pattern look like for you?
- What do you find most satisfying about this shift pattern?
- What do you find most frustrating/challenging about your shift pattern?
- If you could choose an ideal shift pattern, what would be the most important factor that you’d consider in making that choice? Why?
- If we were to change shift patterns, what would be non-negotiable for you?
- Describe your well-being and work-life balance?
- Are there any other comments about your shift pattern?

Close

- Thank the participant for their time.
- Reminder to participants that they can contact you via the study email address provided in the PIS.

**2: Example Group Interview Topic Guide**

*NB: This is an example of the group interview topic guide used during the Improving Shifts for Nursing Staff Qualitative Study. This topic guide was used for NHS Directors in November 2023 to January 2024.*

Overall Study Aim: Our study aims to explore how the preferences and constraints of nursing staff, nurse managers, and hospital directors interact and collectively influence decision-making processes regarding shift patterns.

Opening

- Welcome participants and introduce yourself as the researcher (including name, job role and organisation).
- Ask whether the participant has any questions before we begin.
- Remind participants that they are free to withdraw at any time, and for up to 24 hours following this interview.
- Remind participants that no personal information (such as their name or employer) will be shared with anyone outside the research team.
- Remind the participant that the interview is being recorded and gain consent.
- State that the estimated focus group length will be 60 minutes.
- Outline the purpose of the study and aims of today’s interview.

Main discussion

- What do you consider when planning or considering shift patterns for the nursing staff in your Trust? What outcomes/targets drive your choices?
- What works with the current shift patterns organisation in your Trust?
- What works with the current shift patterns organisation in your Trust?
- If we were to change shift patterns, what would be non-negotiable for you as a Trust manager?
- Are there any other comments about shift pattern organisation?

Close

- Thank the participants for their time.
- Reminder participants that they can contact you via the study email address provided in the PIS.
